# Prenatal environmental tobacco smoke exposure: a predictor of blood selenium levels in children

**DOI:** 10.3389/fpubh.2025.1580316

**Published:** 2025-07-25

**Authors:** Paweł Gać, Michał Fułek, Aleksandra Żórawik, Rafał Poręba, Krystyna Pawlas, Natalia Pawlas

**Affiliations:** ^1^Department of Environmental Health, Occupational Medicine and Epidemiology, Wroclaw Medical University, Wroclaw, Poland; ^2^Department of Diabetology, Hypertension and Internal Diseases, Institute of Internal Diseases, Wroclaw Medical University, Wroclaw, Poland; ^3^Department of Biological and Medical Foundations of Sport, Wroclaw University of Health and Sport Sciences, Wroclaw, Poland; ^4^Department of Pharmacology, Faculty of Medical Sciences in Zabrze, Medical University of Silesia, Katowice, Poland

**Keywords:** active smoking, environmental tobacco smoke, passive smoking, prenatal exposure, selenium blood concentration

## Abstract

**Introduction:**

The aim of the study was to investigate the relationship between prenatal environmental tobacco smoke (ETS) exposure and blood selenium concentration (Se-B) in a selected group of children.

**Methods:**

A total of 299 children were recruited for this study. Prenatal ETS exposure (understood as active as well as passive mother’s exposure) was assessed among all the study participants using a standardized exposure scale. The participants were tested for Se-B.

**Results and discussion:**

Se-B (μg/L) was statistically significantly lower in the group of children with prenatal exposure to ETS compared to those without prenatal exposure (74.35 ± 12.45 vs. 78.60 ± 11.66, *p* < 0.01). Similarly, children whose mothers actively smoked tobacco during pregnancy exhibited lower Se-B than children whose mothers did not smoke (72.09 ± 14.20 vs. 77.58 ± 11.70, *p* < 0.05), and a similar trend was observed for passive smokers (74.63 ± 12.35 vs. 78.40 ± 11.75, *p* < 0.01). While negative correlations were observed between the severity of ETS exposure and Se-B, these results were not statistically significant. Independent risk factors for lower Se-B included advanced age (Rc: −2.398, *p* < 0.05), body weight deficiency (Rc for lower body mass index within the range of underweight to normal body weight: 0.687, *p* < 0.05), and prenatal ETS exposure (Rc: −4.209, *p* < 0.05). This study highlights the association between maternal tobacco smoke exposure during pregnancy and reduced selenium levels in offspring, emphasizing the importance of targeted interventions in prenatal care to minimize ETS exposure.

## Introduction

Selenium is one of the trace elements with a significant impact on the functioning of the entire human body (1). It plays a critical role in maintaining homeostasis, mainly as a structural component of a wide range of enzymes (2). Selenium is incorporated into amino acid derivatives, which form compounds essential to crucial proteins, such as selenoproteins (3). These selenoproteins play a vital role in redox regulation, immune function, and cellular defense mechanisms, thus supporting overall health (4–7). For instance, a strong relationship has been established between blood selenium concentration (Se-B) and a higher risk of cardiovascular mortality (8–12). Notably, both high selenium levels and insufficient Se-B concentrations are linked to metabolic syndrome (13, 14) or metabolic dysfunction-associated steatotic liver disease (MASLD) (15).

Moreover, selenium is involved in processes such as carcinogenesis, immune response regulation, and reproductive system functioning (16–19). As an antioxidant, selenium is crucial for protecting cells from oxidative stress, which is particularly important in inflammatory processes and during fetal development (17, 20). As a key component of thioredoxin reductase, selenium regulates cellular proliferation and apoptosis (20).

Despite the well-established roles of selenium, there is a notable gap in understanding how prenatal environmental tobacco smoke (ETS) exposure specifically affects selenium levels in children. This study addresses a novel and underexplored area by investigating the link between prenatal ETS exposure and selenium levels, offering valuable insights into an intersection of environmental and nutritional health impacts. While existing research highlights the general impact of ETS and selenium deficiencies on health, studies directly linking prenatal ETS to blood selenium concentrations in children are scarce. Addressing this gap can provide new insights into the combined impact of environmental exposures and trace elements on early childhood health outcomes.

Exposure to environmental tobacco smoke (ETS) is an urgent issue in environmental medicine (21). Despite significant advancements in understanding the dangers of ETS exposure since the late twentieth century, public awareness remains insufficient (22). ETS refers to any tobacco smoke exposure outside of active smoking, encompassing second-hand smoke (SHS) and third-hand smoke (THS) (23). Although smoke-free laws have been implemented in many countries, less than 22% of the global population is fully protected by comprehensive smoking bans in public spaces (24).

Maternal smoking during pregnancy is a modifiable yet critical risk factor for low birth weight and preterm birth (25). Studies increasingly suggest that children of mothers who smoked during pregnancy have a higher susceptibility to cardiovascular and metabolic diseases later in life (26–29). However, despite the known harmful effects of maternal smoking, the potential impact of passive ETS exposure during pregnancy on micronutrient status in offspring remains insufficiently explored. Prenatal exposure to ETS may interfere with selenium metabolism by increasing the demand for antioxidant defenses and disrupting the placental transport of essential micronutrients. Cigarette smoke is a potent source of reactive oxygen species (ROS) and nitrogen species (RNS), which can increase oxidative stress and deplete antioxidant defenses (30), including selenium-dependent enzymes. Although studies have primarily focused on active smoking, prenatal ETS exposure, particularly passive exposure during pregnancy, is also a critical source of oxidative stress in the developing fetus (31).

The scientific debate regarding optimal selenium intake remains ongoing (20, 32–34). Since the first recommendation in 1957 (35), the most often cited requirement values are in the range of 20–70 μg (32, 34, 36, 37) or 40–50 μg Se per day (38). European Food Safety Authority (EFSA) instituted adequate Se daily intake as 70 μg for adults and 85 μg for lactating women (39). Tolerable upper intake of Se was established by the Institute of Medicine of the National Academy of Sciences of the United States as 400 μg per day for adults (40).

In the literature there is a surprising number of studies supporting the idea that selenium can either ameliorate or harm human health depending on the dose (41–45). There remains considerable uncertainty regarding the ideal serum selenium levels, as various studies have yielded inconsistent findings (46, 47). The uncertainty over the definition of optimal selenium status seems to be the main reason for the difference between dietary recommendations for selenium in various countries (48–51). In fact, the way of measuring the impact of selenium intake on Se-B is troublesome itself. Current dietary recommendations are mostly based on the activity of glutathione peroxidase, which in turn does not seem to be an appropriate endpoint. In a randomized, double-blind, placebo-controlled dietary intervention performed by Hurst et al. (52), the Plasma selenoprotein P was found to be a useful biomarker of selenium status, as it responds to different dietary forms of selenium.

The aim of this study was to examine the relationship between prenatal ETS exposure and blood selenium concentration in children residing in industrial areas of the Silesian Voivodeship in Poland. By identifying this link, the study underscores the broader significance of understanding how environmental exposures during pregnancy can impact essential micronutrient levels, which are critical for the long-term health and development of children. By providing evidence of this relationship, we hope to inform public health policies and prenatal care strategies aimed at minimizing ETS exposure during pregnancy, ultimately contributing to improved health outcomes for children.

## Methods

Children were recruited from classes of preliminary education of primary school located in industrial area in Upper Silesia, Poland. This was a cross-sectional study and that participants were recruited using convenience sampling from selected schools. 365 children were invited to participate in the study. The final database included 299 subjects (the participation rate was 82%).

Group size was determined using a sample size calculator. The selection conditions were as follows: population size 200,000 (the size of the population of early school children in the Upper Silesian Voivodeship in Poland), fraction size 0.25 (the percentage of smokers in the population of this region), maximum error 5%, confidence level 95%. The required minimum size of the study group was 290.

The mean age in the group amounted to 7.92 years, the mean height was 1.32 m, the mean body mass was 29.82 kg, and the mean body mass index (BMI) was 17.01 kg/m^2^. The examined children were healthy. Selenium supplementation was not used in the study group. General characteristics of the investigated group of children is shown in Table 1.

**Table 1:** Principal clinical characteristics of the studied group of children.

Parameter	X	X_G_	SD	Min	Max
Age (years)	7.92	7.88	0.86	5.00	11.00
Height (m)	1.32	1.31	0.08	1.05	1.60
Body mass (kg)	29.82	28.85	8.00	14.00	62.00
BMI (kg/m^2^)	17.01	16.75	3.08	8.54	28.96
	*n*		%		
Male gender	165		55.2		
Female gender	134		44.8		

BMI, body mass index; Max, maximal value; Min, minimal value; *n*, number; SD, standard deviation; X, arithmetic mean; XG, geometric mean.

Among all children included in the study, a survey analysis was conducted using an original questionnaire. The information about sex of the child, age, family income, mother’s and father’s education, as well as the child’s medical history, development and habits, and exposure to tobacco smoke (ETS) was obtained basing on this questionnaire which was completed by the parents of the children.

Body height and mass were measured. BMI was calculated using the equation: BMI = body mass/height^2^, in which body mass was expressed in kg and body height in meters.

Exposure to tobacco smoke was characterized based on 7 questions of the questionnaire regarding: prenatal exposure to ETS, active tobacco smoking by mother during pregnancy, number of cigarettes smoked daily by the mother during pregnancy (n/day), mother’s passive exposure to ETS during pregnancy, number of hours of the passive exposure to ETS of mother during pregnancy (n/day), current exposure to ETS and number of hours of current passive exposure to ETS (n/day).

The survey form was validated by conducting a re-examination in a randomly selected sample of approximately 10% of the surveys, obtaining similar results.

The participants were tested for blood selenium concentration (Se-B). Hydride generation atomic absorption technique was performed to determine the Se-B. For the digestion of blood samples the mixture of nitric acid/perchloric acid: 7/3 was used. This process was performed under the conditions of standard temperature. The completion of digestion was obtained within 16 h with the maximum temperature of 225°C. The reduction of Se from VI to IV was achieved by adding the 2.5 M hydrochloric acid and warming the samples up to 110°C for 30 min. The UNICAM continuous vapor generation system was used to analyze the samples after dilution. The calibration was accomplished by the usage of calibration standards, which were prepared in advance in the mixture of perchloric acid and hydrochloric acid solution.

Mean Se-B were calculated for the entire group and for subgroups distinguished considering prenatal exposure to ETS, maternal active tobacco smoking during pregnancy, mother’s passive exposure to ETS during pregnancy and current exposure to ETS.

Statistical analysis was conducted using the “STATISTICA 13” software (StatSoft Polska). For quantitative variables geometric means (XG), arithmetic means (X), standard deviations (SD) and range (Min and Max values) of examined parameters were established for each group and subgroup. Distribution of the variables was examined using tests of Lilliefors and W-Shapiro–Wilk. In view of the abnormal distributions, in further analysis testing of hypotheses involving equality of means in two subgroups employed the non-parametric U test of Mann–Whitney, while testing of hypotheses in three or more numerous subgroups took advantage of the nonparametric equivalent of analysis of variance, the test ANOVA of Kruskal–Wallis. Statistically significant differences between arithmetic means were determined using the *post hoc* test of Tukey. Results for qualitative (nominal) variables were expressed in absolute values. For independent qualitative variables, the chi-square test was used for further statistical analysis. Relationships between studied variables were determined using analyses of correlation and backward stepwise multivariate regression analysis. Due to the abnormal distribution of variables, correlation coefficients r of Spearman was determined. The dependent variable in the multivariate regression analysis was the blood selenium concentration (Se-B), and the potentially independent variables were age, gender, body mass, BMI, and variables characterizing ETS. The parameters of the model obtained in the regression analysis were estimated using the least squares method. Results at the *p* < 0.05 level were accepted to be statistically significant.

## Results

Se-B concentrations in the studied group of children amounted to 76.93 ± 12.08 μg/L. Normative blood selenium concentration (Se-B ≥ 80 μg/L) characterized only 33.8% of the investigated population. The characteristics of exposure to ETS and the status of blood selenium concentration in the studied group are summarized in Table 2.

**Table 2:** Exposure to ETS and Se-B in investigated group of children.

Parameter	*n*		%		
Prenatal exposure to ETS	124		41.5		
Active tobacco smoking by mother during pregnancy	39		13.0		
Mother’s passive exposure to ETS during pregnancy	114		38.1		
Current exposure to ETS	79		26.4		
Selenium deficiency (Se-B < 40 μg/L)	0		0.0		
Threshold low Se-B (Se-B: 40–59 μg/L)	18		6.0		
Suboptimal Se-B (Se-B: 60–79 μg/L)	147		49.2		
Optimal Se-B (Se-B ≥ 80 μg/L)	101		33.8		
	X	X_G_	SD	Min	Max
Number of cigarettes smoked daily by the mother during pregnancy (*n*/day)	8.86	7.28	5.44	2.00	20.00
Number of hours of the passive exposure to ETS of mother during pregnancy (*n*/day)	4.72	3.46	3.94	0.50	20.00
Number of hours of current passive exposure to ETS (*n*/day)	3.10	3.05	2.68	0.25	12.00
Se-B (μg/L)	76.93	76.00	12.08	51.00	130.00

ETS, environmental tobacco smoke; *n*, number; Se-B, blood selenium concentration.

Analysis of blood selenium concentration demonstrated a statistically significant difference between subgroup of children prenatal exposed to ETS and subgroup of children prenatal unexposed to ETS, respectively, 74.35 ± 12.45 μg/L vs. 78.60 ± 11.66 μg/L, *p* < 0.01. The above-mentioned difference was observed in active as well as passive exposure to ETS during pregnancy (respectively for mothers with active exposure during pregnancy: 72.09 ± 14.20 μg/L vs. 77.58 ± 11.70 μg/L, *p* < 0.05 and for mothers with passive exposure during pregnancy: 74.63 ± 12.35 μg/L vs. 78.40 ± 11.75 μg/L, *p* < 0.01). Comparative analysis of blood selenium concentration failed to demonstrate any significant differences between subgroups of children distinguished based on current exposure to ETS. Children with current exposure and without current exposure to ETS manifested statistically equal Se-B (75.34 ± 11.61 μg/L vs. 77.49 ± 12.24 μg/L, *p* > 0.05). The status of blood selenium concentration in the studied subgroups distinguished based on the characteristics of exposure to ETS is summarized in Table 3.

**Table 3:** Blood selenium concentration (μg/L) in subgroups divided based on the characteristics of ETS exposure.

Differentiation criterion	Subgroup	*n*	Se-B (μg/L)	Threshold low Se-B (%)	Suboptimal Se-B (%)	Optimal Se-B (%)
Prenatal exposure to ETS	YES	124	74.35 ± 12.45	10.9	56.4	32.7
	NO	175	78.60 ± 11.66	4.1	55.5	40.4
	*p*		<0.01	ns	ns	ns
Maternal active tobacco smoking during pregnancy	YES	39	72.09 ± 14.20	21.9	50.0	28.1
	NO	260	77.58 ± 11.70	4.9	56.3	38.7
	*p*		<0.05	<0.01	ns	<0.05
Mother’s passive exposure to ETS during pregnancy	YES	114	74.63 ± 12.35	9.7	57.3	33.0
	NO	185	78.40 ± 11.75	5.2	53.9	40.9
	*p*		<0.01	ns	ns	ns
Current exposure to ETS	YES	79	75.34 ± 11.61	8.4	53.5	38.0
	NO	220	77.49 ± 12.24	6.4	56.1	37.4
	*p*		ns	ns	ns	ns

ETS, environmental tobacco smoke; ns, statistically insignificant; Se-B, blood selenium concentration.

Statistically non-significant correlations were found between the quantitative variables characterizing exposure to ETS and Se-B. The results of the correlation analysis are summarized in Table 4.

**Table 4:** Results of correlation analysis in the studied group of children.

Parameter	Se-B (μg/L)
Number of cigarettes smoked daily by mother during pregnancy (n/day)	*r* = − 0.15 / ns
Number of hours of the passive exposure to ETS of mother during pregnancy (n/day)	*r* = − 0.16 / ns
Number of hours of current exposure to ETS (n/day)	*r* = − 0.05 / ns

ETS, environmental tobacco smoke; ns, statistically insignificant; Se-B, blood selenium concentration.

In the investigated group of children, the independent risk factors for a lower Se-B represented: a more advanced age, lower BMI values (with BMI according to the percentile charts falling within the range of underweight to normal body weight) and prenatal exposure to ETS. The results of the regression analysis are summarized in Table 5.

**Table 5:** Results of the analysis of the final model obtained in backward stepwise multivariate regression analysis in the investigated group of children.

Model for: Se-B (μg/L) ^a^				
Regression model evaluation parameter	Intercept	Age (years) ^b^	BMI (kg/m^2^) ^b^	Prenatal exposure to ETS ^b, #^
Regression coefficient	86.262	−2.398	0.687	−4.209
SEM of Rc	8.163	0.957	0.268	1.692
*p* value	<0.001	<0.05	<0.05	<0.05
*p*	<0.01			

Risk factors for Se-B. #, dichotomous variable, where 1 – no, 2 – yes; BMI, body mass index; ETS, environmental tobacco smoke; a, dependent variable; b, independent variable; Rc, regression coefficient; Se-B, blood selenium concentration; SEM, standard error mean.

**Figure 1:**
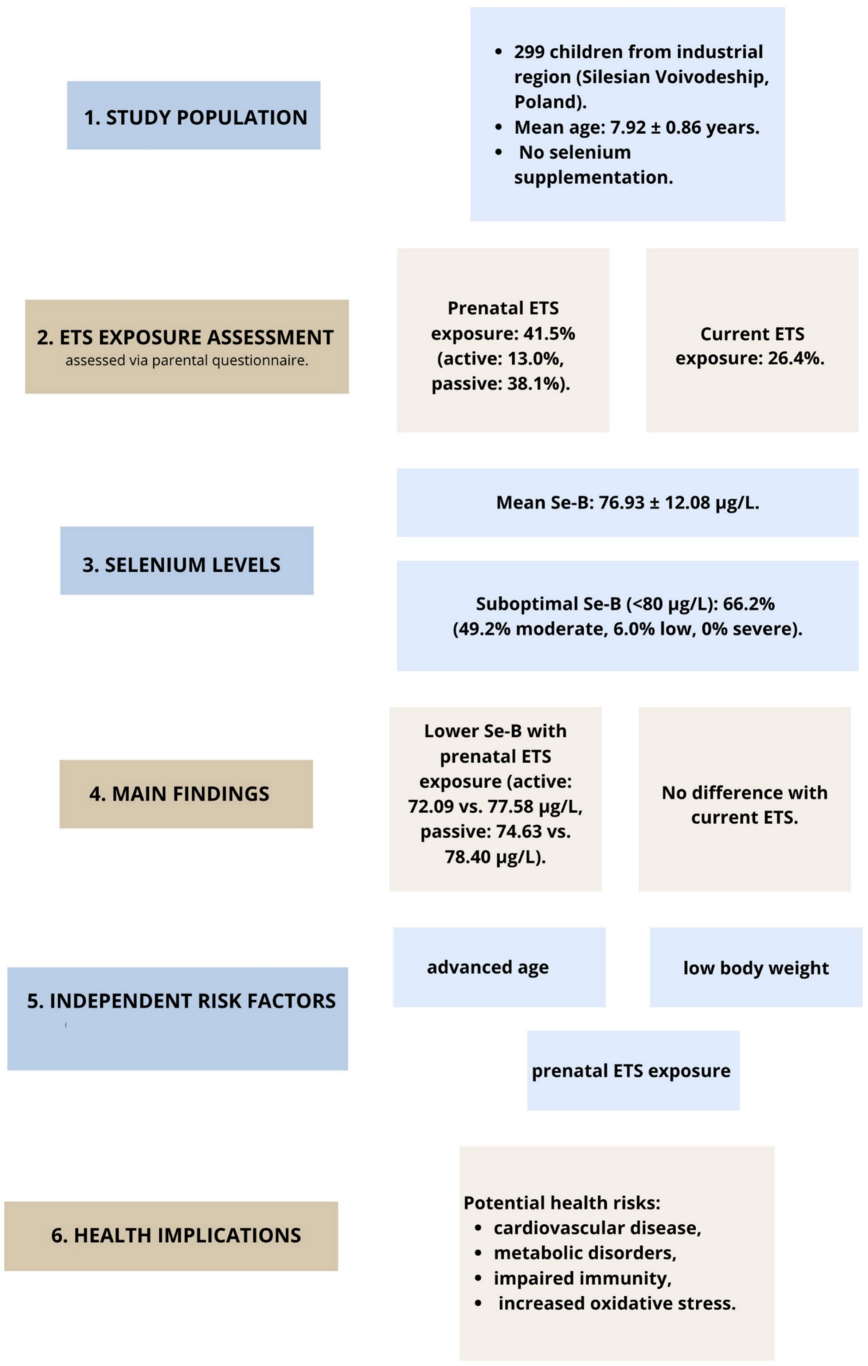
Flow chart summarizing the study population, exposure characteristics, selenium levels, main findings, independent risk factors, and potential health implications.

A summary of the key findings, including ETS exposure characteristics, selenium levels, identified risk factors, and potential health implications, is presented in Figure 1.

## Discussion

There is much evidence to support the hypothesis that low Se status is correlated with an elevated risk of several diseases. These include among others: malignant tumors, thyroid and neurological impairments, infectious diseases, mood disorders and cardiovascular diseases (53–56). The available studies justify the statement that environmental tobacco smoke exposure needs to be regarded as an independent risk factor of suboptimal blood selenium concentration (57–59). The results obtained in our study, however, additionally emphasize the novel discovery – the influence of prenatal smoke exposure. Future research should further develop and confirm these initial findings.

To our knowledge, there has been little discussion on the direct impact of passive or active smoking on Se-B. There is already plenty of evidence however supporting the idea that smoking-induced reactive oxygen- and nitrogen species (ROS and RNS) result in oxidative stress in inflammation and carcinogenesis (59). While Se keeps playing a vital role in oxidative stress (60) as an antioxidant of protective properties (61), it is undeniably one among many components afflicted by tobacco smoke.

However, interestingly, the discrepancy in findings across studies, such as those by Pizent et al. (62) and Liu et al. (63), may reflect differences in study design, sample size, or measurement techniques. While some studies found no association between ETS exposure and selenium status, others, including our own, suggest that prenatal exposure to ETS could lead to reduced selenium levels, potentially due to increased oxidative stress and selenoprotein consumption.

Prior research suggests that oxidative stress induced by tobacco smoke may increase the utilization of selenium-dependent antioxidant enzymes, such as glutathione peroxidase (GPx), leading to the depletion of circulating selenium levels. This is particularly concerning during fetal development, as selenium plays a crucial role in fetal growth and immune system development. Further studies are required to clarify the precise biological pathways through which prenatal ETS exposure disrupts selenium metabolism and to identify potential interventions that could mitigate these effects.

However, interestingly, Pizent et al. (62) conducted a study on trace element-dependent enzyme concentrations (among others Se-GPx (glutathione peroxidase (GPx) dependent on selenium (Se) present in the cytoplasm and the mitochondria)) in maternal and neonatal plasma in a group of smoking and non-smoking mothers and found no difference in selenium-dependent enzyme concentrations between the groups. Selenium concentrations in maternal and fetal serum were also compared, with the result of significantly lower selenium concentrations in the fetal sample, regardless of the mother’s tobacco smoking history. An association between passive exposure to tobacco smoke and maternal plasma selenium concentration was also not observed in a study conducted by Liu et al. (63). However, different results were obtained by Sun et al. (64). It has been demonstrated that due to the consumption of selenoproteins as antioxidants in oxidative stress, Se levels are low in pregnant women exposed to secondhand smoke. Discrepancies in the test results obtained may be due to limitations of the sample size and/or measurement accuracy.

Arnaud et al. (65) showed that smoking habits were inversely related to Se-B. According to van den Brandt et al. (66) however, an inverse association between Se-B was observed with active smoking but not with smoking in the past. Kocyigit et al. (67) compared a group of adult non-smokers with those with at least a 10-year history of active smoking and described the change in antioxidative enzyme activity depending on their cofactor concentrations in tobacco smokers. According to obtained results, plasma selenium concentration and erythrocyte glutathione peroxidase activities were significantly lower in tobacco smokers than in non-smokers. Aycicek et al. (68) conducted research on the effect of passive exposure to ETS among infants on micronutrients concentrations, however, its subjects were iron, zinc and copper, and selenium concentration dependence was not studied. A decrease in iron and zinc concentrations and no difference in copper concentrations in infants exposed to secondhand smoke were described.

We found that the independent risk factors for a lower Se-B included body weight deficiency and more advanced age. Our data suggest that both lower and higher BMI values may be associated with reduced selenium levels in children, potentially indicating a non-linear, U-shaped relationship. However, we emphasize that this observation is preliminary and based on a relatively small sample size. Therefore, it should be interpreted with caution and regarded as hypothesis-generating. Further research in larger and more diverse populations is needed to verify the existence of such a pattern and to explore the underlying mechanisms, which may involve nutritional status, metabolic demands, or differential selenium utilization across weight categories. Similarly, according to a study by Conner et al. (69), individuals with higher selenium deficiency were more likely to have lower BMI and be older, but also to be male and non-European. Other studies have reported associations between excessive BMI and lower selenium concentrations. Awasthi et al. (70) observed decreased selenium levels in overweight, obese, and severely obese schoolchildren and adolescents in India. Ortega et al. (71) described excessive BMI as a factor associated with lower plasma selenium concentrations in a group of Madrid schoolchildren aged 8–13 years. Navia et al. (72) also found a statistically significant negative correlation between BMI and serum selenium (*r* = −0.396; *p* < 0.001). The inverse correlation between age and selenium concentration in the general population has also been well documented (49, 63, 73, 74).

In light of our findings, several preventative strategies could be considered for integration into prenatal care guidelines to better protect maternal and child health. Educational programs targeting pregnant women and their families may help raise awareness of the risks associated with ETS exposure during pregnancy. In addition, routine screening for active and passive tobacco exposure, combined with tailored counseling interventions, should be implemented in antenatal settings. Promoting smoke-free environments—both at home and in shared public spaces—remains a critical public health objective (75). These strategies, if systematically applied, could contribute to the reduction of prenatal ETS exposure and its potential adverse effects, including alterations in micronutrient status such as selenium levels. Emphasizing such preventative approaches aligns with current efforts to improve perinatal outcomes and reduce health disparities related to environmental exposures.

Although our study adds novel evidence regarding the inverse association between prenatal ETS exposure and selenium levels in children, we acknowledge that its observational design precludes direct exploration of biological mechanisms. The lack of mechanistic data represents a limitation in fully elucidating the pathways involved. Nevertheless, prior research suggests that oxidative stress induced by tobacco smoke may increase the utilization of selenium-dependent antioxidant enzymes, such as glutathione peroxidase (GPx) (76), thereby depleting circulating selenium levels. Moreover, fetal exposure to ETS could potentially interfere with trace element transport and metabolism during critical stages of development. Future studies incorporating biomolecular analyses—such as markers of oxidative stress, inflammation, or selenoprotein activity—are needed to clarify these mechanistic links and deepen our understanding of how prenatal environmental exposures affect selenium bioavailability.

The strength of the investigation conducted is that it is a study emphasizing yet undiscovered influence of prenatal passive tobacco smoke exposure on blood selenium concentration in children but are aware that our research has some limitations. In terms of material, the main limitation of the study is the relatively small number of patients included. Further studies involving a larger sample group are needed to confirm our research results. In terms of methodology, to assess the environmental smoke exposure, we used a survey method. Therefore, it can be suspected that, consciously or not, some respondents may have provided misleading answers due to an inaccurate awareness of their addiction or a reluctance to admit to a habit commonly perceived as harmful to health. Self-reports may also be subject to recall bias or underreporting, as individuals might inaccurately disclose their smoking habits due to social desirability or a lack of awareness about the extent of their exposure. While this approach was chosen as the most feasible method within the scope of our study, we recognize that it may not capture the full complexity of ETS exposure. Future studies could benefit from incorporating biomarkers such as cotinine levels to provide a more objective measure of exposure, complementing self-reported data. A similar problem was described by Benedetti et al. (77). The authors point out that data collected through a questionnaire filled out by study participants does not necessarily coincide with the truth, as they tend to describe their unhealthy addiction on a smaller scale than it is (77). Overall, the data show trends of underestimating smoking prevalence when based on self-report compared to an estimation of smoking prevalence based on cotinine concentrations (78–81). Conducting a similar study in the future using cotinine concentration to determine actual exposure to tobacco smoke could improve the accuracy and repetitiveness of the results obtained. The lack of data on nutrition should also be pointed out as a significant limitation of the study.

## Conclusion

Our study demonstrated a significant association between prenatal exposure to environmental tobacco smoke (ETS) and reduced blood selenium concentration in children, with the findings emphasizing the impact of maternal tobacco exposure during pregnancy on critical micronutrient levels. The strength of this association was evident in the statistical analyses, which indicated that prenatal ETS exposure serves as an independent risk factor for suboptimal selenium levels.

The implications of our findings are particularly relevant in the context of the established health risks associated with selenium deficiency, including its effects on immune function, oxidative stress regulation, and long-term metabolic health. By identifying prenatal ETS exposure as a contributing factor to these deficiencies, our study highlights a crucial area for public health intervention.

This new understanding underscores the importance of public health policies aimed at reducing tobacco smoke exposure during pregnancy. Educational campaigns targeting expectant mothers and stricter regulations to minimize ETS exposure could have profound implications for improving maternal and child health outcomes.

We recommend that future research further investigates the mechanisms linking ETS exposure to selenium metabolism and evaluates the potential benefits of selenium supplementation in mitigating the adverse effects of prenatal ETS exposure. Additionally, integrating these findings into prenatal care guidelines could enhance preventative strategies and support better health for both mothers and their children.

## Data Availability

The raw data supporting the conclusions of this article will be made available by the authors, without undue reservation.
